# Investigations on Nuclear DNA Content and DNA Synthesis in Plants and Fungi Using Flow Cytometry and Fluorescence Microscopy

**DOI:** 10.3390/genes13030417

**Published:** 2022-02-25

**Authors:** Elwira Sliwinska

**Affiliations:** Laboratory of Molecular Biology and Cytometry, Department of Agricultural Biotechnology, Bydgoszcz University of Science and Technology, Kaliskiego Ave. 7, 85-796 Bydgoszcz, Poland; elwira@pbs.edu.pl

The twenty-first century has been an era of extensive genome exploration and modifications, using advanced methods such as genome sequencing and editing. Information on genome size (nuclear DNA content) and DNA synthesis has become a required basis for underpinning these advances. However, genome size data are available only for approximately 12,000 plant species (Kew Plant DNA C-values Database (https://cvalues.science.kew.org/, accessed on 12 January 2022), e.g., in the family Orchidaceae, which contains about 30,000 species, genome size is known merely for 1.5% of them [1]. Therefore, there is an urgent need for further measurements. The most widely used method applied to establish nuclear DNA content in plants and fungi is flow cytometry (FCM). It is fast, accurate, convenient, and relatively inexpensive. In addition to using FCM for measuring genome size, it is also applied for establishing plant ploidy, DNA base composition, and the intensity of endoreduplication and cell cycle (for a recent review on applications and best practices in plant FCM, see Sliwinska et al. [2]). In some applications, FCM is supplemented by cytogenetic methods such as microscopic counting of chromosomes and/or in situ hybridization with the use of fluorescence microscopy.

In this Special Issue, there are nine papers covering different applications of FCM and fluorescence microscopy in plants. Establishing genome size, an important species characteristic, can be helpful in species identification, particularly during early plant development when it is difficult to differentiate between related species based on morphological characteristics. Rewers et al. [1] determined the 2C DNA content of 15 species and one intraspecific taxon of wild orchids growing in Poland. FCM revealed differences in genome size between 12 of the species, which is significant enough to distinguish them. For the identification of the remaining species, additional applications of molecular markers or sequencing are required. Analysis of genome size is also often used in research on plant evolution. In order to reveal the evolutionary history of sweet vernal grass (*Anthoxanthum odoratum*), Chumová et al. [3] combined FCM, molecular analyses of chloroplast and nuclear DNA, and fluorescence and genomic in situ hybridization. They established the nuclear DNA content of 103 populations of *A. odoratum* and eight populations of *A. alpinum* located throughout Europe and found large intraspecific variations in *A. odoratum*, which correlate with latitude and altitude. The results confirm the allopolyploid origin of this species and an autopolyploid origin of tetraploid *A. alpinum*. Polyploidy played an important role also in the evolution of the genus *Festuca*. FCM and chromosome counting were applied to establish genome size and ploidy of two subspecies of *Festuca yvesii* (ssp. *summilusitana* and *lagascae*) collected from 13 populations on the Iberian Peninsula [4]. This study reveals a polyploid series at the subspecies level, including 12*x* and 14*x* cytotypes. Interestingly, although nuclear DNA content increased with ploidy levels, this increase was not proportional (the monoploid genome size decreased), which confirms genome downsizing during polyploidization of *Festuca*. Polyploidization also accompanied speciation in the *Echeveria* genus [5]. Nuclear DNA content and chromosome number were established for 23 *Echeveria* species collected in Mexico, revealing the presence of 2*x*, 4*x*, 5*x*, 6*x*, and 10*x* accessions as well as two hexaploid-aneuploid species; genome size varied from 1.26 pg/2C to 7.70 pg/2C, and monoploid genome size, similarly to *Festuca*, correlated negatively with ploidy levels.

In addition to plant systematics and evolution, genome size/ploidy estimation is applied to plant breeding, especially in polyploid and hybrid breeding. Due to the fact that polyploids are usually characterized by larger organs than diploids, they produce higher biomass and increased secondary metabolites; therefore, they are desired in agriculture. Moreover, polyploidy often reduces fertility of plants, which is a beneficial trait in some breeding programs, e.g., infertility of hemp prevents yield reduction caused by pollination. Crawford et al. [6] used colchicine treatments in vitro to produce tetraploids from two *Cannabis sativa* inbred lines and then, after crossing plants of different ploidies, obtained diploid (2*x*), triploid (3*x*), and tetraploid (4*x*) hybrids. Indoor and field trials were performed to assess several plant phenotypic traits. To render the experiments reliable, the ploidy of parents and F_1_ hybrids was estimated using FCM and chromosome counting. Infertile triploid hybrids were obtained, with characteristics of higher biomass, inflorescence weight, and cannabinoid concentrations compared to diploids, which makes them promising as a material to create enhanced hemp cultivars.

New traits can be also introduced to commercial cultivars by interspecific hybridization. However, success in crossing plants of different species, even those related, is usually difficult because of genetic incompatibility. In such breeding programs, knowledge of the genome sizes of potential parents can be helpful in predicting cross-compatibility. With the aim of supporting breeding programs of *Geranium*, Akbarzadeh et al. [7] estimated genome size, chromosome number, and genetic relationship between 61 different *Geranium* species and commercial cultivars. They recommend the pairwise Jaccard similarity coefficient as a preliminary determinator for successful hybrid production in this genus.

An important requirement for plant breeding is the collection of germplasm for genetic improvement of the cultivars/lines. Such collections are also useful for research, e.g., in the conservation of diverse ecosystems. The accessions of such collections have to be well characterized, including plant genome size/ploidy and reproduction modes. These traits can be estimated by using FCM. Grasslands and rangelands are the largest ecosystems and also are feed resources for livestock. One of the important forage grasses group belongs to the genus *Urochloa*. Tomaszewska et al. [8] undertook research aimed at optimizing FCM procedures by using dry leaf material, instead of the usual fresh leaves, for the estimation of ploidy of 348 *Urochloa* accessions from the United States Department of Agriculture (USA), the Vavilov Research Institute (Russia), and the Centro Internacional de Agricultura Tropical (Colombia) germplasm collections; some were included in breeding programs. In order to validate their FCM results, chromosomes were counted using epifluorescence microscopy. The results reveal that FCM enables robust ploidy estimation of these tropical forage grasses from dehydrated specimens using described methods for sampling, drying, storage, transportation, preservation, and sample preparation. The presented protocol is of great importance, because it allows estimations of ploidy of plants in wild geographical areas, where fresh leaves cannot be analyzed due to the lack of flow cytometers.

FCM is a unique method that is applied to study endoreduplication (a process in which nuclear DNA amplification is not followed by mitosis, which results in somatic polyploidy—endopolyploidy—of some differentiated cells). Although this is very common in plants, its physiological significance is still unclear and requires further studies. Palomino et al. [5] estimated the endoreduplication pattern in leaves of some species of *Echeveria* adapted to different environments to show a relationship between endopolyploidy level and stress caused by environmental changes. Depending on the species, the maximum level of endopolyploidy varied between 8C and 64C, and it was suggested that endoreduplication is a defense mechanism triggered by various types of stresses. Changes in endoreduplication intensity during seed development makes the measurement of endopolyploidy suitable for estimating seed maturity, and it can also be related to stress conditions. Nowicka et al. [9] estimated endopolyploidy in different tissues (embryo, endosperm, and maternal tissues) of wild barley grains originating from mesic, semi-mesic, semi-xeric, and xeric ecogeographic sites in Israel collected at different developmental stages from 4 to about 65 days after pollination. According to the obtained results, in wild barley grains, endoreduplication is higher than those of cultivated barley, and the proportion of endoreduplicated nuclei in the endosperm is higher in xeric accessions. This again suggests that endoreduplication is an adaptive mechanism to harsh environmental conditions.

In order to improve understanding of the role of endoreduplication in the biology and evolution of mosses, Pal’ová et al. [10] performed FCM on different parts and developmental stages of eleven moss species, nine collected from naturally growing populations and two cultured in vitro. They observed higher endopolyploidy in gametophytes than in sporophytes; moreover, within cauloids and phylloids, it is higher in the basal regions than in the apical regions. Similarly to higher plants, higher endopolyploidy occurs in ontogenetically older parts of mosses.

The information presented by the authors in this Special Issue will enrich the Plant DNA C-values Database with new estimations of genome size and broaden our knowledge of the physiological and adaptive roles of endoreduplication in plant development. My thanks goes to all authors and reviewers for their contributions to this Special Issue “Investigations on Nuclear DNA Content and DNA Synthesis in Plants and Fungi Using Flow Cytometry and Fluorescence Microscopy.” I am sure you will join me in looking forward to learning more as research on these important topics continues.

## References

[1] Rewers M., Jedrzejczyk I., Rewicz A., Jakubska-Busse A. (2021). Genome Size Diversity in Rare, Endangered, and Protected Orchids in Poland. Genes.

[2] Sliwinska E., Loureiro J., Leitch I.J., Šmarda P., Bainard J., Bureš P., Chumová Z., Horová L., Koutecký P., Lučanová M. (2021). Application-Based Guidelines for Best Practices in Plant Flow Cytometry. Cytom. Part A.

[3] Chumová Z., Mandáková T., Trávníček P. (2021). On the Origin of Tetraploid Vernal Grasses (*Anthoxanthum*) in Europe. Genes.

[4] Martínez-Sagarra G., Castro S., Mota L., Loureiro J., Devesa J.A. (2021). Genome Size, Chromosome Number and Morphological Data Reveal Unexpected Infraspecific Variability in *Festuca* (Poaceae). Genes.

[5] Palomino G., Martínez-Ramón J., Cepeda-Cornejo V., Ladd-Otero M., Romero P., Reyes-Santiago J. (2021). Chromosome Number, Ploidy Level, and Nuclear DNA Content in 23 Species of *Echeveria* (Crassulaceae). Genes.

[6] Crawford S., Rojas B.M., Crawford E., Otten M., Schoenenberger T.A., Garfinkel A.R., Chen H. (2021). Characteristics of the Diploid, Triploid, and Tetraploid Versions of a Cannabigerol-Dominant F1 Hybrid Industrial Hemp Cultivar, *Cannabis sativa* ‘Stem Cell CBG’. Genes.

[7] Akbarzadeh M., Van Laere K., Leus L., De Riek J., Van Huylenbroeck J., Werbrouck S.P.O., Dhooghe E. (2021). Can Knowledge of Genetic Distances, Genome Sizes and Chromosome Numbers Support Breeding Programs in Hardy Geraniums?. Genes.

[8] Tomaszewska P., Pellny T.K., Hernández L.M., Mitchell R.A.C., Castiblanco V., de Vega J.J., Schwarzacher T., Heslop-Harrison P. (2021). Flow Cytometry-Based Determination of Ploidy from Dried Leaf Specimens in Genomically Complex Collections of the Tropical Forage Grass *Urochloa* s. l.. Genes.

[9] Nowicka A., Sahu P.P., Kovacik M., Weigt D., Tokarz B., Krugman T., Pecinka A. (2021). Endopolyploidy Variation in Wild Barley Seeds across Environmental Gradients in Israel. Genes.

[10] Pal’ová M., Ručová D., Goga M., Kolarčik V. (2021). Spatial and Temporal Patterns of Endopolyploidy in Mosses. Genes.

